# A 31‐year‐old with idiopathic reversible cerebral vasoconstriction syndrome

**DOI:** 10.1002/ccr3.1090

**Published:** 2017-07-20

**Authors:** Ihtesham A. Qureshi, Mohtashim A. Qureshi, Obiajulu Kanu, Salvador Cruz‐Flores

**Affiliations:** ^1^ Neurology Department Texas Tech University Health Sciences Center 4800 Alberta Avenue El Paso Texas 79905; ^2^ Internal Medicine Department Texas Tech University Health Sciences Center 4800 Alberta Avenue El Paso Texas 79905

**Keywords:** Cerebral angiogram, migraine, reversible cerebral vasoconstriction syndrome, thunderclap headache

## Abstract

In our patient with reversible cerebral vasoconstriction syndrome (RCVS) syndrome, presenting with thunderclap‐like headache, there is a possibility to be readily confused with migraine. Initiating treatment with selective serotonin reuptake inhibitors (SSRIs) and triptans can further aggravate the condition. Therefore, it is essential to understand the nature and type of headache and correlate the clinical findings with imaging studies.

A 31‐year‐old female presented with severe headache that started a week ago not relieved on pain medications, comes to the hospital with severe headache, intermittent, pulsatile, throbbing type, located over the frontal region bilaterally radiating to the back of the head associated with three episodes of non‐bloody, non‐bilious vomiting. Computed tomography (CT) of head without contrast, lumbar puncture, and CT angiogram head were negative **(**Figs 1 and 2). Secondary causes for reversible cerebral vasoconstriction syndrome (RCVS) like subarachnoid hemorrhage (SAH) were ruled out from negative computed tomography head (CT) and lumbar puncture (LP), while vasculitis was excluded from the laboratory findings that showed normal erythrocyte sedimentation rate (ESR), anti‐nuclear antibody (ANA), anti‐neutrophil cytoplasmic antibody (ANCA) P&C, cryoglobulin, complement, rheumatoid factor (RF), anti SS‐A and SS‐B levels along with unremarkable cerebrospinal fluid analysis (CSF). The patient was started on sertraline and sumatriptan with no relief. Neurology was consulted to evaluate the headache. They recommended to stop both medications and initiate with magnesium, solumedrol, and verapamil 1, 2. Cerebral angiogram was performed and demonstrated the imaging stigmata of RCVS (Fig. 3A and B). Patient's headache improved and was discharged on verapamil with follow up within 4–6 weeks.

**Figure 1:**
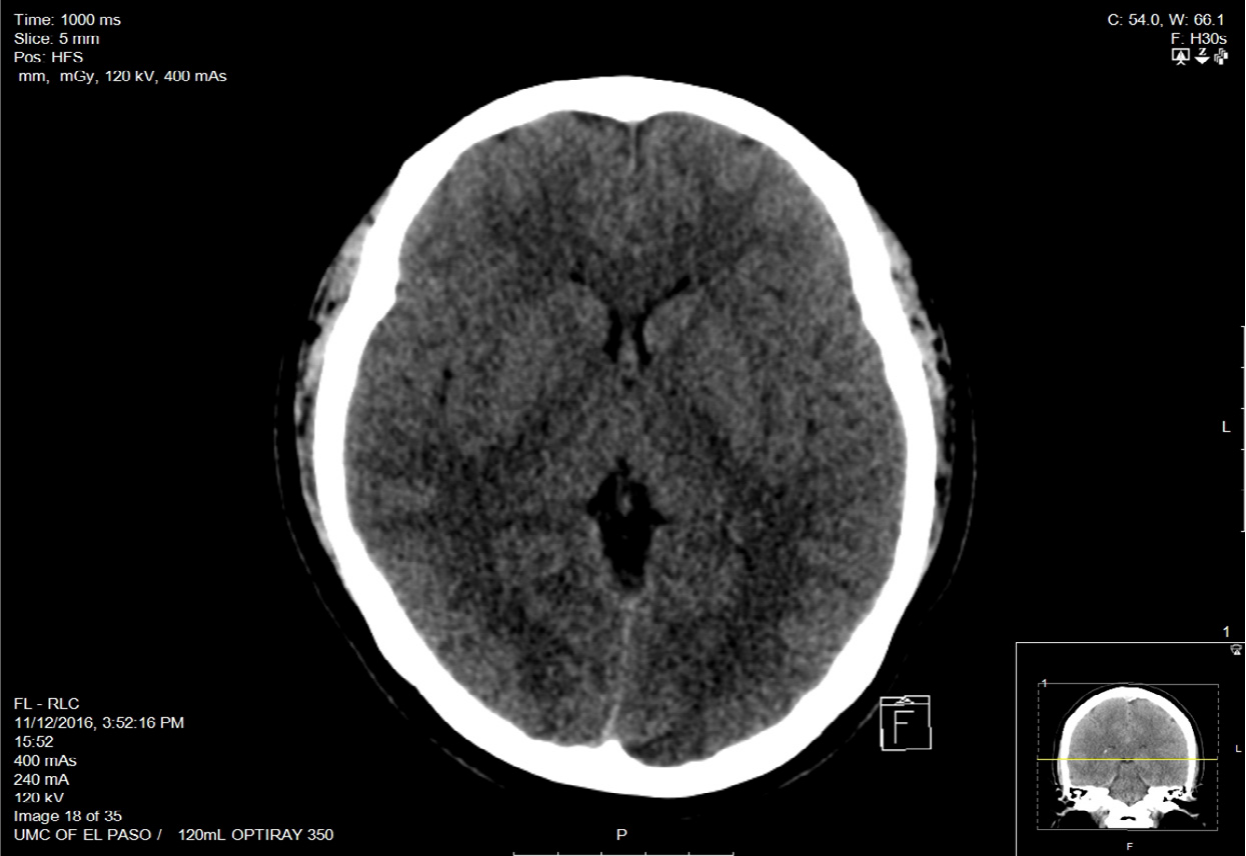
Computed tomography (CT) head and brain without contrast is negative for acute intracranial pathology.

**Figure 2:**
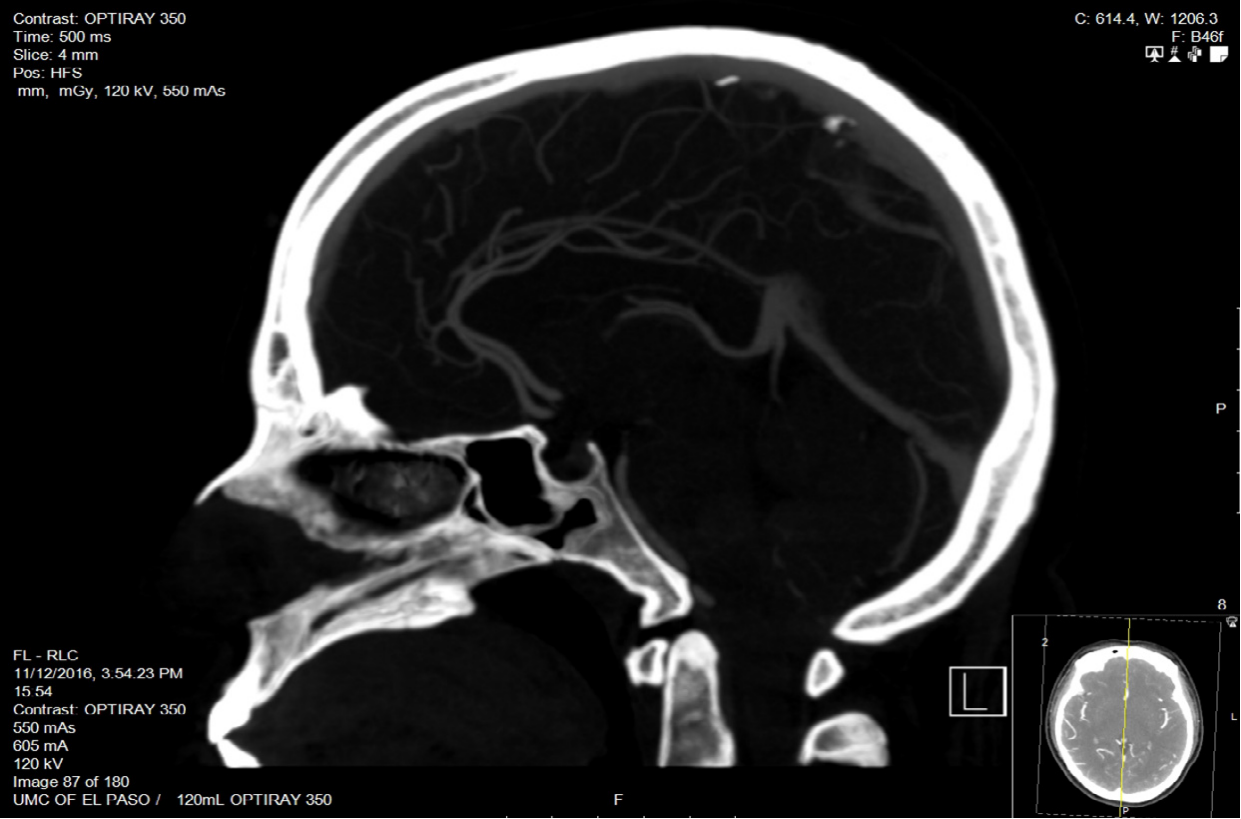
Computed tomography (CT) angiogram with IV contrast was negative for acute intracranial abnormalities, intra‐cranial arterial flow limiting stenosis, aneurysms, dissections, occlusion, or vascular malformations.

**Figure 3:**
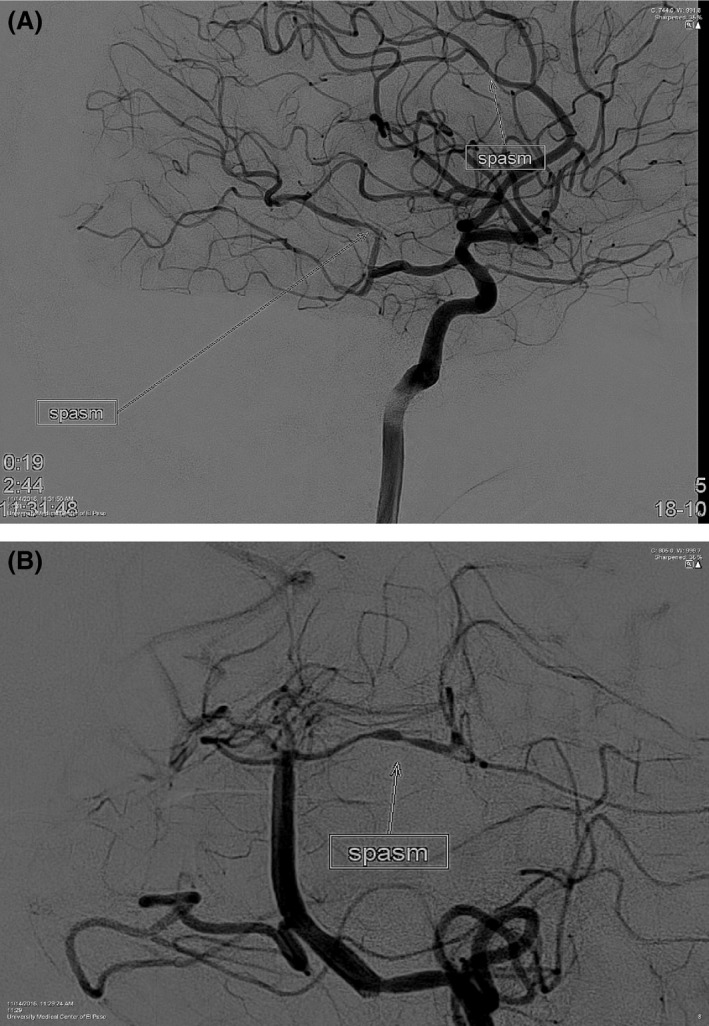
(A) Cerebral angiogram shows multiple areas of arterial constriction in the anterior and posterior circulations findings consistent with reversible cerebral vasoconstriction syndrome. (B) Cerebral angiogram shows multiple areas of arterial constriction in the anterior and posterior circulations findings consistent with reversible cerebral vasoconstriction syndrome.

## Conflict of Interest

None declared.

## Authorship

IAQ: involved in manuscript writing; MAQ: involved in critical revision of the manuscript; OK and SCF: involved in patient care.
